# Conservation of Major Satellite DNAs in Snake Heterochromatin

**DOI:** 10.3390/ani13030334

**Published:** 2023-01-17

**Authors:** Artem Lisachov, Alexander Rumyantsev, Dmitry Prokopov, Malcolm Ferguson-Smith, Vladimir Trifonov

**Affiliations:** 1 Animal Genomics and Bioresource Research Unit (AGB Research Unit), Faculty of Science, Kasetsart University, 50 Ngamwongwan, Chatuchak, Bangkok 10900, Thailand; 2 Institute of Cytology and Genetics, Russian Academy of Sciences, Siberian Branch, Novosibirsk 630090, Russia; 3 Institute of Molecular and Cellular Biology, Russian Academy of Sciences, Siberian Branch, Novosibirsk 630090, Russia; 4 Cambridge Resource Centre for Comparative Genomics, Department of Veterinary Medicine, University of Cambridge, Cambridge CB3 0ES, UK

**Keywords:** Serpentes, Colubridae, Viperidae, sex chromosomes, repetitive DNA, centromere

## Abstract

**Simple Summary:**

In the present work, we describe the satellite DNA families that occur in the genomes of two snakes from different families: *Daboia russelii* (Viperidae) and *Pantherophis guttatus* (Colubridae). We show high conservation of nucleotide sequences and chromosomal localizations of these satellites, despite the widespread view that such genomic elements evolve very rapidly.

**Abstract:**

Repetitive DNA sequences constitute a sizeable portion of animal genomes, and tandemly organized satellite DNAs are a major part of them. They are usually located in constitutive heterochromatin clusters in or near the centromeres or telomeres, and less frequently in the interstitial parts of chromosome arms. They are also frequently accumulated in sex chromosomes. The function of these clusters is to sustain the architecture of the chromosomes and the nucleus, and to regulate chromosome behavior during mitosis and meiosis. The study of satellite DNA diversity is important for understanding sex chromosome evolution, interspecific hybridization, and speciation. In this work, we identified four satellite DNA families in the genomes of two snakes from different families: *Daboia russelii* (Viperidae) and *Pantherophis guttatus* (Colubridae) and determine their chromosomal localization. We found that one family is localized in the centromeres of both species, whereas the others form clusters in certain chromosomes or subsets of chromosomes. BLAST with snake genome assemblies showed the conservation of such clusters, as well as a subtle presence of the satellites in the interspersed manner outside the clusters. Overall, our results show high conservation of satellite DNA in snakes and confirm the “library” model of satellite DNA evolution.

## 1. Introduction

Repetitive DNA sequences are a key component of eukaryotic genomes. There are several types of repeats, classified by their structure and sub-chromosomal localization. Interspersed and tandem repeats are recognized by their genomic organization. Interspersed repeats can be located in various regions of the genome, whereas tandem repeats are mostly organized into clusters in specific segments of chromosomes [1]. Satellite DNA sequences (satDNA) are among the most abundant types of tandem repeats. They are usually located in the C-positive heterochromatic blocks at centromeres, as well as in the pericentromeric, subtelomeric, and, more rarely, interstitial chromosomal regions [2]. Every eukaryotic genome usually contains several families of satDNAs, with each family having its specific localization. For example, centromeric heterochromatin is typically composed of the special centromeric satellite, whereas the pericentromeric heterochromatin blocks harbor the satellites of other families [3]. Some satDNA families and subfamilies occur at similar positions in all chromosomes (e.g., pan-centromeric repeats), whereas others are accumulated on a subset of chromosomes or even one specific chromosome (for example, a sex chromosome) [4]. Specific satellite families spread inside chromosomes and between chromosomes by means of ectopic recombination, gene conversion, and transposition with mobile genetic elements (TEs) [5,6,7]. 

Since satDNAs do not encode proteins, they were once viewed as “selfish”, “junk DNA”, and “genomic parasites”. However, there is a growing body of evidence that satDNA clusters are technical elements of chromosomes that participate in regulating their structure and behavior during the cell cycle, i.e., condensation, decondensation, kinetochore formation, and meiotic pairing [8,9,10]. Depending on their function, satDNAs differ in their degree of conservation. While certain families are species-specific, others can be characteristic for the whole genus or taxonomic family [11,12,13]. It has been hypothesized that satDNA divergence may contribute to the constrained meiotic chromosome pairing in hybrids, thus directly affecting speciation [14]. This makes satDNA an important marker to study phylogenetics, genome evolution, and genome function in diverse animal groups.

In reptiles, satDNAs are poorly studied. A notable exception is the lizard family Lacertidae, in which numerous satellites have been identified and extensively studied [15,16,17,18,19]. Two satellites have been identified in Scincidae [20,21], and two satellite families have been found in Varanidae [22,23]. Four types of satDNAs are known from the Chinese softshell turtle (*Pelodiscus sinensis*) [24]. Recently, a high conservation of tandem repetitive DNAs has been demonstrated in crocodilians [25]. Snakes comprise nearly half of the total squamate diversity; however, data on their satDNAs are scarce. Four families of satDNAs were found in different snake species. The PFL-MspI satellite was isolated from *Protobothrops flavoviridis* (Crotalinae, Viperidae), located in the centromeric regions of its chromosomes. This satDNA is shared at least by *Gloydius blomhoffi* from the same subfamily Crotalinae, as shown by FISH and slot blot hybridization. The slot blot analysis did not reveal this satellite even in *Bitis arietans* (Viperinae, Viperidae), a member of the same family. The PBI-MspI satellite was found in *Python bivittatus*, *P. molurus*, and *Boa constrictor* by FISH and slot blot, indicating the conservation of this satellite at least at the Henophidia level. Lastly, the PBI-DdeI satellite was initially identified as a major centromeric satellite in *P. bivittatus*, whereas FISH and slot blot failed to detect this satellite in any other genus [26]. However, later the PBI-DdeI was found in a wide set of diverse snake species using PCR. In *Naja kaouthia*, this repeat was accumulated in the W chromosome [27]. Apparently, sequence divergence and/or low copy number may impede the detection of a satDNA by hybridization methods. Another repetitive sequence, BamHI-B4, is specific to the terminal part of the homolog of the *Anolis* chromosome 6 (ZZ/ZW chromosome in Caenophidia and XX/XY chromosome in *Python*) and is conserved in pythons, colubrids, and pit vipers [28].

Classical “wet” methods of satDNA isolation include the analysis of genomic fragments in gradient centrifugation and the digestion of genomic DNA with restriction enzymes, while a range of bioinformatic approaches have recently been suggested to search for tandemly arranged DNAs in genomic data. In the present work, we used the Tandem Repeat Analyzer software (TAREAN) [29] to identify satellite repeats in two species of snakes, *Daboia russelii* (Viperinae, Viperidae) and *Pantherophis guttatus* (Colubridae), from short genomic reads. This software de novo identifies tandem organized satellite repeats from raw Illumina reads of a genomic sample. We studied their chromosomal localization using FISH and analyzed the cross-species conservation using BLAST on the available snake genome assemblies. The genome assemblies of *Vipera latastei* (Viperinae, Viperidae) (rVipLat1.pri) and *V. ursinii* (rVipUrs1.1) were used for quantitative and localization analysis, since they have the best assembled repeat clusters among the available assemblies of snakes.

## 2. Materials and Methods

### 2.1. Cell Line Establishment and Karyotype Analysis

The *P. guttatus* and *D. russelii* cells were grown from fibroblasts obtained from the Cambridge Resource Center for Comparative Genomics, Department of Veterinary Medicine, UK. The cell cultures were provided to the Institute of Molecular and Cellular Biology, SB RAS, Russia for joint research. The cell lines of *P. guttatus* and *D. russelii* were deposited in the IMCB SB RAS cell bank (“The general collection of cell cultures”, 0310-2016-0002). Chromosome suspensions from the cell cultures were obtained in the Laboratory of Comparative Genomics, IMCB SB RAS, Novosibirsk, Russia, as described previously [30,31].

### 2.2. Repetitive DNA Identification 

DNA sequencing data were downloaded from the NCBI SRA database (accession number SRR5506741 for *D. russelii* genomic reads and SRR9596755 for *P. guttatus*) and used for the identification of tandemly arranged repeats. Filtering by quality and adapter trimming was performed using fastp 0.23.2 [32] with the parameters “--detect_adapter_for_pe -5 -3 -r -l 75”. Trimmed reads were used in the analysis with the TAREAN 2.3.7 tool [29], which identified clusters of the most abundant tandemly arranged repeats. NCBI BLAST [33] was used to compare consensus tandem repeat sequences with available genome assemblies. RepBase was used to compare consensus tandem repeat sequences with available described repeat sequences [34] 

### 2.3. Fluorescence In Situ Hybridization (FISH) 

DNA of *P. guttatus* and *D. russelii* was extracted from the cell cultures using the standard phenol–chloroform technique. Primers for PCR amplification and labeling of seven probes were designed with PrimerQuestTool [35] (Table 1). PCR amplification was performed as described earlier [36]. Labeling was performed using PCR by incorporation of biotin-dUTP and digoxigenin-dUTP (Sigma, Darmstadt, Germany). FISH was performed in accordance with previously published protocols [37]. Images were captured using the VideoTest-FISH software (Imicrotec, New York, NY, USA) with a JenOptic charge-coupled device (CCD) camera (Jena, Germany) mounted on an Olympus BX53 microscope (Shinjuku, Japan). All images were processed in Adobe PhotoShop 2021 (Adobe, San Jose, CA, USA).

## 3. Results

### 3.1. Tandem Repeat Identification

The TAREAN analysis revealed four high-confidence satellite repeats in the genome of *D. russelii* and three high-confidence satellite repeats in the genome of *P. guttatus*, which were named DRU-Sat-1, DRU-Sat-2, DRU-Sat-3, DRU-Sat-5, PGU-Sat-1, PGU-Sat-2, and PGU-Sat-3, respectively (Table 2). The satellites DRU-Sat-1 and PGU-Sat-1 were found to belong to the same family, while the satellites DRU-Sat-2, PGU-Sat-2, and PGU-Sat-3 belonged to another family. Interestingly, DRU-Sat-2 and PGU-Sat-2 shared a high level of similarity and were more distantly related to PGU-Sat-3 (Table 3, File S1).

### 3.2. FISH Analysis

**Figure 1:**
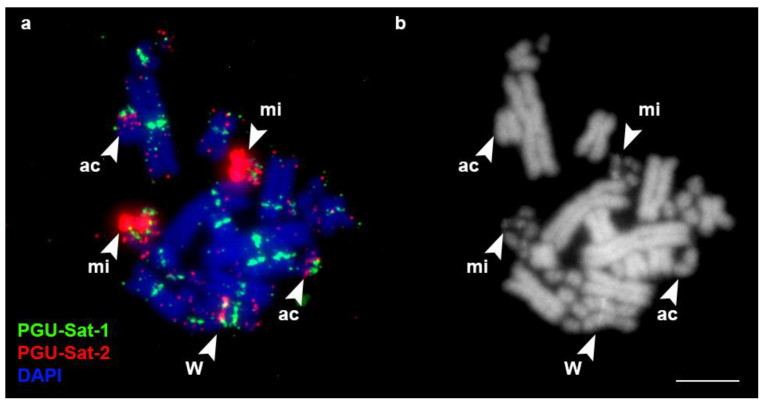
Localization of the satellites PGU-Sat-1 and PGU-Sat-2 in the chromosomes of *P. guttatus.* mi: the PGU-Sat-2 bearing microchromosome; ac: the acrocentric macrochromosome with both PGU-Sat-1 and PGU-Sat-2 signals; W: the W chromosome. (**a**) Merged image; (**b**) DAPI channel. Scale bar: 10 μm.

**Figure 2:**
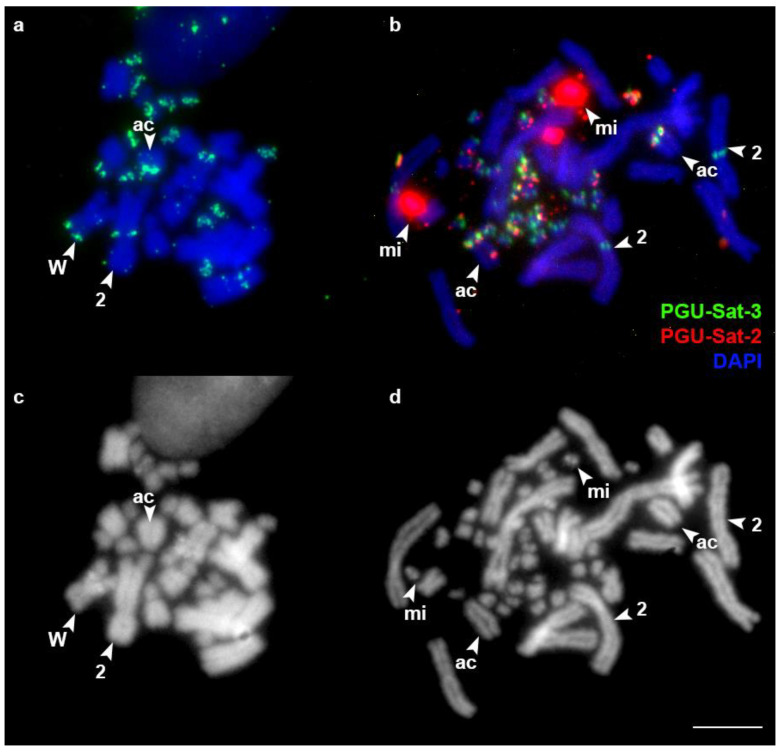
Localization of the satellites PGU-Sat-2 and PGU-Sat-3 in the chromosomes of *P. guttatus.* mi: the PGU-Sat-2 bearing microchromosome; ac: the acrocentric macrochromosome with both PGU-Sat-2 and PGU-Sat-3 signals; 2: chromosome 2; W: the W chromosome. (**a**,**b**) Merged images; (**c**,**d**) DAPI channel. Scale bar: 10 μm.

The karyotypes of the studied specimens comprised 36 chromosomes (eight pairs of macrochromosomes and 10 pairs of microchromosomes) with pairs of heteromorphic Z and W chromosomes. This is a typical snake karyotype that corresponds to the previously described karyotypes of these species [38,39]. In *P. guttatus*, the satellite PGU-Sat-1 was localized in the centromeric regions of macrochromosomes and in several microchromosomes. It was also localized in the DAPI-positive interstitial band of the W chromosome (Figure 1). The PGU-Sat-2 and PGU-Sat-3 satellite types, despite belonging to the same family, showed strikingly different chromosomal localizations. The PGU-Sat-2 satellite was mapped to the same DAPI-positive band in the W chromosome and in the pericentromeric region of one small acrocentric macrochromosome. It was also present in certain pairs of microchromosomes, being extensively amplified in one pair (Figure 1, Figure 2 and Figure S1). The PGU-Sat-3 satellite tended to be localized in microchromosomes, but not in all pairs. It was colocalized with PGU-Sat-2 in the pericentromeric region of one small acrocentric macrochromosome, and it was also present in the pericentromeric region of the q-arm of the chromosome 2 and in the terminal region of the p-arm of the W chromosome (Figure 2).

**Figure 3:**
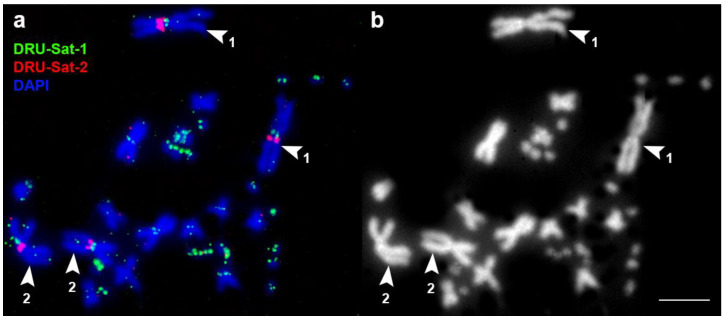
Localization of the satellites DRU-Sat-1 and DRU-Sat-2 in the chromosomes of *D. russelii.* 1: chromosome 1; 2: chromosome 2. (**a**) Merged image; (**b**) DAPI channel. Scale bar: 10 μm.

**Figure 4:**
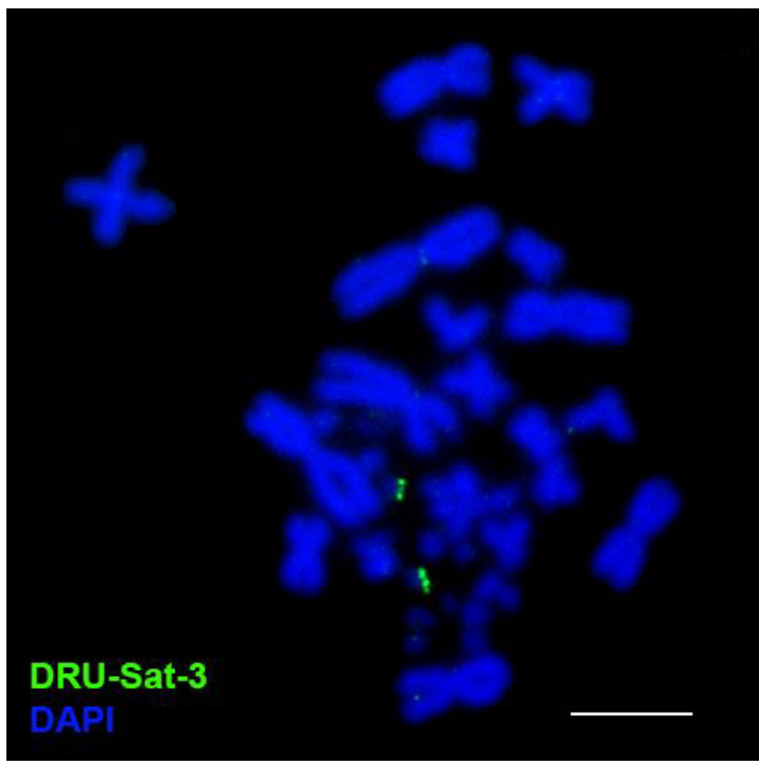
Localization of the satellite DRU-Sat-3 in the chromosomes of *D. russelii.* Scale: 10 μm.

**Figure 5:**
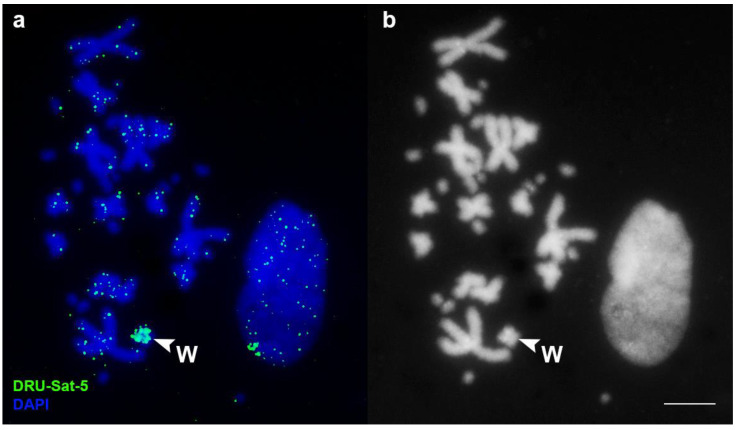
Localization of the satellite DRU-Sat-5 in the chromosomes of *D. russelii*. W: the W chromosome. (**a**) Merged image; (**b**) DAPI channel. Scale bar: 10 μm.

In *D. russelii*, the satellite DRU-Sat-1 was localized in the centromeric areas of all chromosomes. The satellite DRU-Sat-2 was localized in the p-arm of the chromosome 1 and the q-arm of chromosome 2 (Figure 3). The satellite DRU-Sat-3 was chromosome-specific and showed a band in one pair of microchromosomes (Figure 4). The satellite DRU-Sat-5 was amplified throughout the whole length of the W chromosome (Figure 5).

### 3.3. BLAST Analysis

Even though none of the detected satellites were found in the nr/nt NCBI database by BLAST, we found that the DRU-Sat-1/PGU-Sat-1 satellite belongs to the same family as PFL-MspI, which was described earlier [26,27]. We did not reveal homology between the other detected satDNAs and any of the previously described snake repetitive elements. However, we detected all the satellite families found in this work in the RefSeq genomes of other snakes by BLAST. The DRU-Sat-1/PGU-Sat-1, DRU-Sat-2/PGU-Sat-2/PGU-Sat-3 and DRU-Sat-3 satellites were found in various higher snakes, namely, *Protobothrops mucrosquamatus*, *Crotalus tigris* (Crotalinae, Viperidae), *Notechis scutatus*, *Pseudonaja textilis* (Elapidae), *Thamnophis sirtalis*, and *Thamnophis elegans* (Colubridae). Interestingly, BLAST revealed the DRU-Sat-3 satellite in *Pantherophis guttatus*, whereas TAREAN did not. The DRU-Sat-5 satellite was not found in any genome assemblies except those of Viperidae. 

The alignment of DRU-Sat-1/PGU-Sat-1 to the genome assembly of *V. latastei* revealed its high copy number in all chromosome scaffolds except 15 and 17 (from 8877 in scaffold 3 to 358 in the scaffold Z), with predominantly medial localization, possibly corresponding to the centromere. The percentage identity between DRU-Sat-1/PGU-Sat-1 and the *V. latastei* sequences did not vary between the scaffolds and was between 95% and 97% for DRU-Sat-1. 

The DRU-Sat-2/PGU-Sat-2/PGU-Sat-3 satellite was present in the scaffolds 1–3, Z, and 5–10, being the most abundant in scaffolds 2, 3, and 5. The copy numbers were 17,561, 2923, and 2522, respectively, in contrast to 101 in scaffold 1, where it was the second most abundant. In scaffolds 2, 3, and 5, this satellite was accumulated in clusters surrounding the centromere, possibly corresponding to the pericentromeric C bands. The copies in scaffolds 2, 3, and 5 had higher similarity to DRU-Sat-2 than the copies located in the scaffolds where this satellite was less abundant (percent of identity 91.86–94.08% versus 71.97–90.8%). 

The satellite DRU-Sat-3 was present in 983 copies in scaffold 16 and was clustered in the subterminal position of the p-arm, if the DRU-Sat-1/PGU-Sat-1 cluster is considered as the centromere. Scaffold 2, where it was the second most abundant, harbored only 12 copies. The copies located in scaffold 16 had up to 96.88% identity with DRU-Sat-3, whereas the copies from other scaffolds had 75.86–93.1% identity. In the assembly of *V. ursinii,* scaffold 15 with 459 copies and similar cluster localization was the only scaffold where DRU-Sat-3 was found.

Lastly, satellite DRU-Sat-5 showed a small copy number in the genome assembly of *V. latastei* (up to 117 in the scaffold 1) and did not show any clustering. The W chromosome was not present in the assembly. In the assembly of *V. ursinii*, DRU-Sat-5 was accumulated in the W chromosome (1998 copies), whereas, in the autosomes, it had no more than 15 copies per chromosome.

## 4. Discussion

The satDNAs DRU-Sat-1/PGU-Sat-1, DRU-Sat-2/PGU-Sat-2/PGU-Sat-3, and DRU-Sat-3 are found in a wide range of higher snake genomes, which means that they originated at least in the common ancestor of Viperidae and Colubridae at ~42 MYA [40]. In contrast, DRU-Sat-5 is apparently younger as it is restricted to the Viperidae. Since it is present in both Viperinae and Crotalinae, its estimated age is therefore around 31 MY [40]. Previously, a more ancient snake satDNA, PBI-DdeI, which is shared by Henophidia and Caenophidia, was described [27]. In most species, it has a low copy number and probably lacks the tandem organization pattern; therefore, it is detectable only by PCR and BLAST with good-quality genome assemblies, and not by FISH and slot blot [26,27]. This satellite was also not detected by TAREAN in our work, although it is probably present in the genomes of the species studied, since TAREAN detects only highly repeated and tandemly organized elements. Possibly, PBI-DdeI is dispersed and low-copy in the genomes of *D. russelii* and *P. guttatus.* These findings challenge the common conception that satDNAs evolve very rapidly and are usually restricted to one species or a narrow phylogenetic clade, since the “recent appearance” may in fact mean a “recent rise in copy number” of an ancient satellite [41]. According to the concept known as the “library” model of satDNA evolution, animal genomes usually contain many diverse families of satDNAs (the “library”), only a few of which are highly amplified. During phylogenesis and speciation, the “library” experiences dynamic evolution, with satDNA families rising and decreasing in copy number, which leads to contrasting satDNA profiles in related species despite the qualitative conservation of the satDNA repertoires [11].

PGU-Sat-2 strongly indicates a pair of microchromosomes and may represent a considerable part of this chromosomes content. This is in contrast to avian microchromosomes, which are usually gene-rich and heterochromatin-poor. The revealed accumulation makes the PGU-Sat-2 probe a convenient tool for microchromosome identification.

The distribution of BLAST hits of the detected satellites in the genome assemblies of *V. latastei* and *V. ursinii* was similar to that observed in the FISH results for *D. russelii*. Specifically, DRU-Sat-1/PGU-Sat-1, which belong to the same family as the previously described PFL-MspI satellite, represent a centromeric repeat, DRU-Sat-2/PGU-Sat-2/PGU-Sat-3 is located in the pericentromeric clusters in a subset of macrochromosomes, DRU-Sat-3 is accumulated in one pair of microchromosomes, and DRU-Sat-5 is accumulated in the W chromosome. This result indicates that this satellite landscape at least predates the divergence between *Vipera* and *Daboia*, which occurred around 15 MYA [40]. We suppose that PCR for the DRU-Sat-5 marker may serve as a molecular sexing method for at least *Vipera* and *Daboia*. It should be further tested in other species of Viperidae.

## 5. Conclusions

In this work, we described four satellite DNA families in snake genomes, and revealed their chromosomal localization using FISH and BLAST in chromosome-level genome assemblies. Three of these four families are completely novel. We show that three families are conserved in Colubridae and Viperidae, whereas one is characteristic for Viperidae. In two satellite families, the pattern of chromosomal localization is conserved in both Colubridae and Viperidae, and, in two families, it is conserved in *Daboia* and *Vipera.* Our results indicate that, despite the common opinion that satellite DNA evolves extremely quickly and is usually species- or genus-specific, ancient repeat families are not rare. This corroborates the “library” model of the satellite DNA evolution, which supposes that diverse types of satellites may coexist in the genome, and that the common view of their very rapid appearance and disappearance may be due to their changes in copy number.

## Figures and Tables

**Table 1:** Primers used to amplify satDNA in the current study.

Satellite	Primer Sequences
PGU-Sat-1	F 5’–TTTCAAGTACGAGCTTTCCC–3’ R 5’–GCTGAATTGAGCCCTACTG–3’
PGU-Sat-2	F 5’–GACACCAGGATGAGTTTCAG–3’ R 5’–TCCTGACCGTGGAGTAAA–3’
PGU-Sat-3	F 5’–CTTCCTCGGGCAGCAAA–3’ R 5’–GTAACAACGGATGCTAGAATGT–3’
DRU-Sat-1	F 5’–CCCGCCTGACCGAAGACC–3’ R 5’–GAGCTCTATCTGCAACGGG–3’
DRU-Sat-2	F 5’–ACCCCGAATCTCATTCTGGC–3’ R 5’–TCCTGATGCCGGGGTCAG–3’
DRU-Sat-3	F 5’–TTGTGTTTCTGGATCAATAACC–3’ R 5’–GCCTTTCCTGTATAATCCAAA–3’
DRU-Sat-5	F 5’–CAGAGCTGCTGGGAAGTG–3’ R 5’–GAGATCAATGAGGACCCCA–3’

**Table 2:** Putative satellites revealed by TAREAN in the genomes of *Daboia russelii* and *Pantherophis guttatus*.

Sattelite Name	Monomer Size (bp)	Genome Proportion, %	GC	Species	Accession Number
			Content, %		
DRU-Sat-1	168	0.3	42.3	*Daboia russelii* (Russell’s viper)	OP820475
DRU-Sat-2	170	0.13	35.9	—//—	OP820476
DRU-Sat-3	64	0.012	37.5	—//—	OP820477
DRU-Sat-5	147	0.025	44.9	—//—	OP820478
PGU-Sat-1	167	0.31	42.5	*Pantherophis guttatus*	OP820479
				(Corn snake)	
PGU-Sat-2	187	0.085	37.4	—//—	OP820480
PGU-Sat-3	169	0.043	39.1	—//—	OP820481

**Table 3:** The p-distances between the consensus sequences of the PGU-Sat-2/PGU-Sat-3/DRU-Sat-2 family.

	PGU-Sat-2	PGU-Sat-3	DRU-Sat-2
PGU-Sat-2	-	—//—	—//—
PGU-Sat-3	0.278	-	—//—
DRU-Sat-2	0.262	0.226	-

## Data Availability

The consensus sequences of the identified satDNAs are deposited in GenBank.

## Supplementary Materials

The following supporting information can be downloaded at https://www.mdpi.com/article/10.3390/ani13030334/s1: File S1. FASTA alignment file showing sequence similarity of DRU-Sat-2, PGU-Sat-2, and PGU-Sat-3. Localization of the satellite PGU-Sat-2 (red) in the chromosomes of *P. guttatus* with reduced exposure time to show the signal in the PGU-Sat-2-bearing microchromosome in more detail. (a) Merged image; (b) DAPI channel. Scale bar: 10 μm.
