# Supplementary figures and images for: Conservation of Major Satellite DNAs in Snake Heterochromatin
Source: Animals (Basel). 2023 Jan 17;13(3):334. doi: 10.3390/ani13030334 (PMC9913375; doi:10.3390/ani13030334)

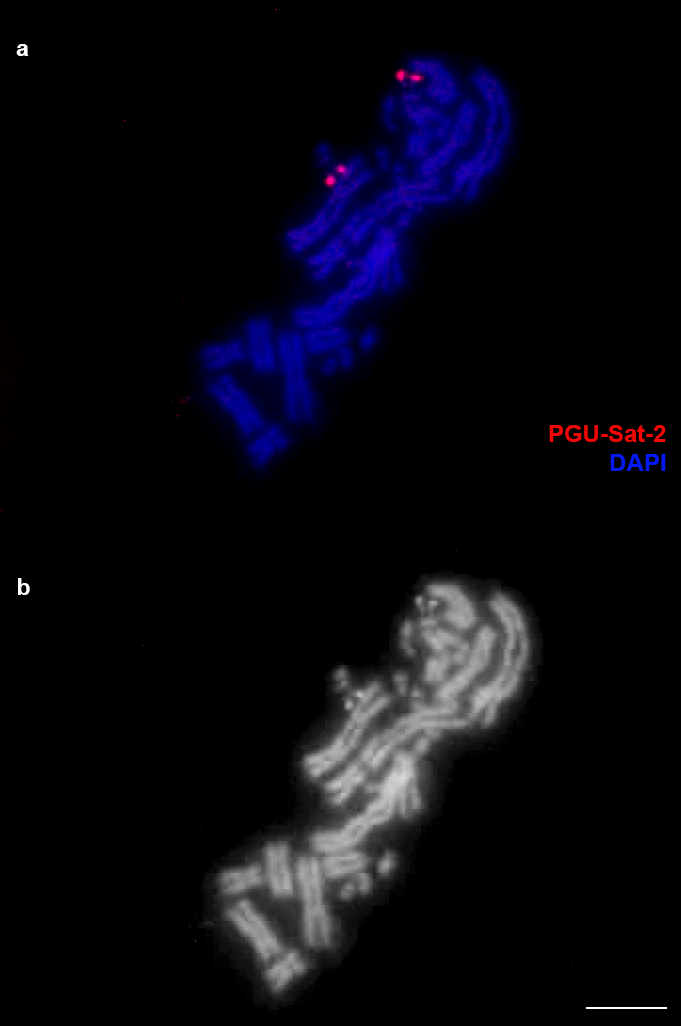

Supplement: Supplementary file 1 [file animals-13-00334-s001.zip › animals-2115624-supplementary/FigureS1.tif]
